# Immune Correlates of Disseminated BCG Infection in IL12RB1-Deficient Mice

**DOI:** 10.3390/vaccines10071147

**Published:** 2022-07-19

**Authors:** Xuyang Wang, Liqiu Jia, Yang Liu, Jing Wang, Chao Qiu, Tao Li, Wenhong Zhang, Zhaoqin Zhu, Jing Wu, Yanmin Wan

**Affiliations:** 1 National Medical Center for Infectious Diseases, Shanghai Key Laboratory of Infectious Diseases and Biosafety Emergency Response, Department of Infectious Diseases, Huashan Hospital, Shanghai Medical College, Fudan University, Shanghai 200040, China; wydasydj@163.com (X.W.); jia895398400@163.com (L.J.); zhangwenhong@fudan.edu.cn (W.Z.); 2 Shanghai Public Health Clinical Center, Department of Laboratory Medicine, Shanghai 201508, China; liuyang656092953@163.com (Y.L.); wangjing_1888@163.com (J.W.); zhaoqinzhu@163.com (Z.Z.); 3 Key Laboratory of Medical Molecular Virology (MOE/MOH), Institutes of Biomedical Sciences, Shanghai Medical College, Fudan University, Shanghai 200032, China; qiuchao@fudan.edu.cn; 4 Shanghai Public Health Clinical Center, Department of Tuberculosis, Shanghai 201508, China; litao@shaphc.org; 5 National Clinical Research Center for Aging and Medicine, Huashan Hospital, Fudan University, Shanghai 200040, China; 6 Shanghai Public Health Clinical Center, Department of Radiology, Shanghai 201508, China

**Keywords:** IL12RB1, MSMD, IFN-γ, BCG, innate immunity, adaptive immunity

## Abstract

Interleukin-12 receptor β1 (IL12RB1)-deficient individuals show increased susceptibilities to local or disseminated BCG infection and environmental mycobacteria infection. However, the low clinical penetrance of IL12RB1 deficiency and low recurrence rate of mycobacteria infection suggest that protective immunity still exists in this population. In this study, we investigated the mechanism of tuberculosis suppression using the IL12RB1-deficient mouse model. Our results manifested that *Il12rb1*^−/−^ mice had significantly increased CFU counts in spleens and lungs, especially when BCG (Danish strain) was inoculated subcutaneously. The innate TNF-a and IFN-γ responses decreased, while the IL-17 responses increased significantly in the lungs of *Il12rb1*^−/−^ mice. We also found that PPD-specific IFN-γ release was impaired in *Il12rb1*^−/−^ mice, but the specific TNF-a release was not compromised, and the antibody responses were significantly enhanced. Moreover, correlation analyses revealed that both the innate and PPD-specific IFN-γ responses positively correlated with CFU counts, whereas the innate IL-12a levels negatively correlated with CFU counts in *Il12rb1*^−/−^ mice lungs. Collectively, these findings proved that the adaptive immunities against mycobacteria are not completely nullified in *Il12rb1*^−/−^ mice. Additionally, our results imply that IFN-γ responses alone might not be able to contain BCGitis in the setting of IL12RB1 deficiency.

## 1. Introduction

IL-12 plays a crucial role in regulating both innate and adaptive immunities [1], of which, downstream signaling through IL-12R is thought to be critical for type 1 immune responses [2]. IL-12R is a heterodimer consisting of IL-12Rβ1 and IL-12Rβ2, which bind to IL-12p40 (IL-12b) and IL-12p35 (IL-12a), respectively [2,3,4]. IL-12Rβ1 is a common chain shared by the IL-12 and IL-23 receptors, and its deficiency can cause profound defects in both IL-12 and IL-23 signaling, which renders the hosts susceptible to intracellular microbe infections [5], such as mycobacteria and salmonella.

The essential role of IL12RB1 in resistance against intracellular bacterial pathogens was recognized through IL-12Rβ1-knockout mice [6] and identification of IL12RB1-deficient individuals [7,8], which collectively showed that IL12RB1 deficiency impaired IFN-γ production, a cytokine thought to be critical for the control of mycobacteria infection [9,10,11,12,13]. In addition, recent studies suggested that the development of Th17 cells (IL-17 producing T cells) [14] and circulating memory Tfh (T follicular helper) cells [15] might also be impaired in IL12RB1-deficient patients. Nonetheless, compared with the studies elucidating the immune regulatory effect of IL-12 [1], relatively little is known about the impact of IL12RB1 deficiency on anti-microbial infection [5]. One of the intriguing phenomena is that, although IL12RB1 deficiency increases the susceptibility to childhood-onset mycobacteriosis and salmonellosis, the recurrence of mycobacterial disease is rare, while the recurrence of salmonellosis is much more frequent [16]. Both BCG inoculation and disease were observed to be effective in preventing subsequent environmental mycobacteriosis [17], suggesting that protective immunity against mycobacteria could still be established in IL12RB1-deficient individuals. Further investigations into this phenomenon will not only expand the understanding regarding the function of IL12RB1, but also provide new clues for delineating the protective immunities against mycobacteria.

To this end, in this study, we first compared both the innate and adaptive immunities between wild-type and *Il12rb1*^−/−^ mice upon inoculating BCG, either intranasally or subcutaneously. Our results showed that the patterns of both innate and adaptive immune responses were obviously changed by *Il12rb1* deletion. Of note, we found that the levels of IFN-γ responses correlated positively with BCG CFU counts, while the levels of IL-12a correlated negatively with BCG CFU counts in *Il12rb1*^−/−^ mice, which implied that IFN-γ might not be beneficial for the in vivo containment of BCGitis in the setting of IL12RB1 deficiency.

## 2. Materials and Methods

### 2.1. Ethical Statement

Mice experiments were carried out at Shanghai Public Health Clinical Center (Shanghai, China). All animal experiment protocols were reviewed and approved by Research Ethics Review Committee of the Shanghai Public Health Clinical Center, affiliated with Fudan University (Reference number: 2021-A023-03).

#### 2.1.1. Mice and BCG

*Il12rb1* gene knockout C57BL/6 (*Il12rb1*^−/−^) mouse was constructed by Cyagen Biosciences (Suchow, China) and maintained in the SPF animal facility of Shanghai Public Health Clinical Center. Wild-type C57BL/6 mice were purchased from SLAC Laboratory Animal Co., Ltd. (Shanghai, China). All mice used in this study were female and 8–12 weeks old. The BCG (Danish strain, obtained from Shanghai Biological Products Institute) was kindly gifted by Dr. Honghai Wang from Fudan University (Shanghai, China).

#### 2.1.2. BCG Inoculation and Sample Collection

Female adult C57BL/6 and *Il12rb1*^−/−^ mice were inoculated with BCG either intranasally or subcutaneously (Table 1). For intranasal inoculation, the mice were anesthetized briefly with isoflurane inhalation, and then 1.5 × 10^6^ CFUs of BCG suspended in 50 µL sterile PBS were instilled into both nostrils of mouse noses. For subcutaneous inoculation, 3 × 10^6^ CFUs of BCG suspended in 100 µL sterile PBS were injected under the lateral skin of mouse abdomen. After inoculation, the mice were weighed and monitored every 3 days. On day 27, all the mice were euthanized by cervical dislocation, and peripheral blood, spleen, and lung tissues were collected separately for BCG CFU count and immune assays. Since the genetic background of the mice was homogenous, except that *Il12rb1* gene was negated in *Il12rb1*^−/−^ mice, 5–6 mice per group should be enough to support the statistical analysis. The dosage of BCG used in this study was relatively high because it was intended to be used as a challenge model, rather than a vaccination model. Despite this, the dosages (1.5 × 10^6^ CFUs/mouse for intranasal inoculation and 3 × 10^6^ CFUs/mouse for subcutaneous inoculation) used in our study were still within the reasonable range, as a higher dosage (5 × 10^6^ CFUs/mouse) was used in a previous study [18]. Euthanasia and detection were performed at 4 weeks post inoculation because the BCG load should have dropped dramatically in wild-type mice at this time point [19,20], and the difference between the wild-type and *Il12rb1*^−/−^ mice could be readily observed.

#### 2.1.3. CFU Counts of BCG in Lung and Spleen

After euthanasia, mouse lung and spleen tissues were collected and homogenized using a tissuelyser (Cat# SCIENTZ-48, SCIENTZ, Ningbo, China). The homogenized mixtures were then serially diluted with sterile 1× PBS; then, 100 µL of each diluted sample was spread on a 7H11 agar plate with four antimicrobials (amphoterincin B 10 mg/L, polymyxin B 200,000 units/L, carbenicillin 50 mg/L, and trimethoprim 20 mg/L). To ensure the accuracy of the CFU count, duplicated plates were spread for each sample. The plates were incubated at 37 °C for 3 weeks before counting.

#### 2.1.4. Real-Time PCR Detection of Cytokine Transcription in Lung Tissue

The left lower lobes of lungs were preserved in RNA protect liquid, and the total RNA was extracted using the RNeasy mini kit (Cat# 74104, Qiagen, Hilden, Germany), according to the manufacturer’s protocol. Reverse transcriptions of total RNA were performed using the HiScriptIII RT SuperMix for qPCR (+gDNA wiper) kit (Cat# R323-01, Vazyme, Nanjing, China). Primers for *tnf-a*, *il-1*, *il-2*, *cxcl-2*, *myd88*, *ifn-γ*, *il-17*, *il-4*, *il-6*, *il-12a*, *il-12b*, *il-10*, *inos*, *hif-1a*, and *gapdh* were synthesized by Sangon Biotech (Shanghai, China) (Table S1). Real-time PCR mix was prepared according to the manufacturer’s instruction (TB Green Premix Ex Taq II kit, Cat# RR820A, Takara, China). PCR reactions were run for 40 cycles using the ABI 7500 Real-Time PCR system (Cat# 4351105, ABI, Foster city, CA, USA) under the following conditions: 95 °C pre-denaturation for 30 s, 1 cycle; 95 °C denaturation for 5 s, 60 °C annealing, extension for 30 s, and 40 cycles. *Gapdh* gene was used as an internal control. According to a previous study [21], *gapdh* is one of the top 10 constantly expressed housekeeping genes, which are expressed in multiple mouse tissues (including the lung tissue). The qPCR primers used in this study were synthesized, according to open databases and previous reports [22]. We assume that the amplification efficiencies for all the tested genes are comparable, while calculating the delta delta CT. The transcription levels were calculated according to the following formula: 2^−∆CT^ = 2^Ct value of GAPDH –Ct value of the cytokine^ [23].

#### 2.1.5. Measurement of PPD-Specific IgG in Mouse Serum

An in-house ELISA protocol was developed using purified PPD (purified protein derivative of *M. tuberculosis* H37Rv) protein (Cat# DAG2684, ABACE-BIOLOGY, Beijing, China) as coating antigen. Experiment procedure was established according to our previous work [24].

#### 2.1.6. IFN-γ ELISPOT Assay

Antigen-specific release of IFN-γ was measured using a mouse IFN-γ ELISPOT kit (BD Bioscience, Cat# 551083). Mouse splenocytes and peritoneal macrophages were freshly isolated and seeded into each well at 2 × 10^5^ cells/well and 1 × 10^5^ cells/well, respectively. The cells were stimulated with PPD (at a final concentration of 5 μg/mL) for 20 h in a 37 °C/5% CO_2_ incubator. Spots representing IFN-γ producing cells were enumerated by using an automated ELISPOT plate reader (ChampSpot III Elispot Reader, Saizhi, Beijing, China).

#### 2.1.7. Cytokine Beads Array (CBA) Assay

The concentration of TNF-a, IL-2, IL-17A, and IL-21 in the culture supernatant of IFN-γ ELISPOT assays were measured using a cytokine beads array (CBA) kit (Cat# 558266, BD Bioscience, San Jose, CA, USA). Briefly, 50 µL of supernatant or cytokine standard was mixed with 50 µL detection beads and incubated in dark for 1 h at room temperature. Subsequently, 50 µL PE labeled detection reagent was added to each sample and incubated in dark for 2 h at room temperature. Finally, the beads were washed with 1 mL wash buffer and resuspended in 300 µL wash buffer. Flow cytometry analyses were performed using LSR Fortessa (BD, Franklin lakes, NJ, USA).

#### 2.1.8. Statistical Analysis

All statistical analyses were performed using GraphPad Prism 9 (GraphPad Software, Inc., La Jolla, CA, USA). The distribution of the data was verified by the method of Shapiro–Wilk test. Comparisons between two groups were conducted by the methods of parametric *t*-test for normally distributed data and non-parametric *t*-test for non-normally distributed data. Correlation analyses were done by Pearson correlation (for normally distributed data) or Spearman’s rank correlation (for non-normally distributed data). Heatmap clustering analysis was conducted using an online tool (Heatmapper, www.heatmapper.ca) (clustering method: complete linkage; distance measurement method: Spearman rank correlation). *p* < 0.05 was considered as statistically significant.

## 3. Results

### 3.1. IL12RB1 Deficiency Compromised the In Vivo Containment of BCG Especially When Inoculated Subcutaneously

**Figure 1:**
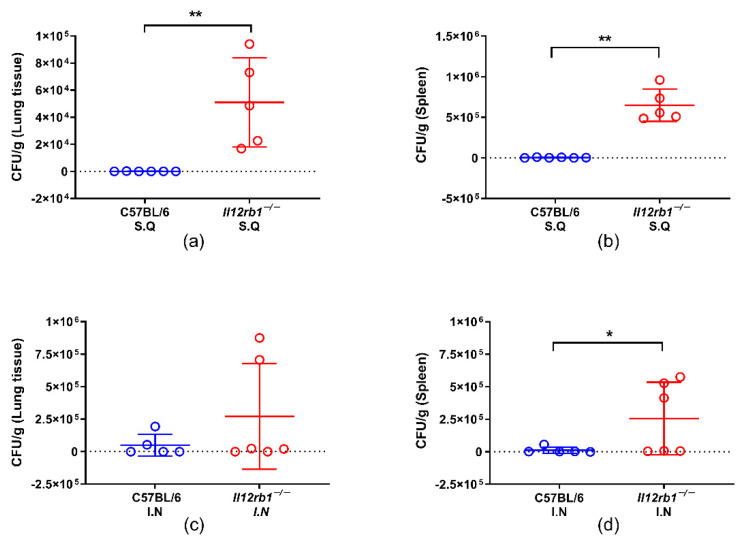
Comparisons of BCG bacterial burden in lungs and spleens of mice. The mice were euthanized 4 weeks after BCG inoculation. The lungs and spleens were harvested and homogenized for the CFU count of BCG. (**a**) Comparison of CFU counts in lungs of *Il12rb1*^−/−^ (*n* = 5) and C57BL/6 mice (*n* = 6) inoculated with BCG subcutaneously. (**b**) Comparison of CFU counts in spleens of *Il12rb1*^−/−^ (*n* = 5) and C57BL/6 mice (*n* = 6) inoculated with BCG subcutaneously. (**c**) Comparison of CFU counts in lungs of of *Il12rb1*^−/−^ (*n* = 6) and C57BL/6 mice (*n* = 5) inoculated with BCG intranasally. (**d**) Comparison of CFU counts in spleens of of *Il12rb1*^−/−^ (*n* = 6) and C57BL/6 mice (*n* = 5) inoculated with BCG intranasally. Statistical analyses were performed by the two-tailed *t*-test method (*, *p* < 0.05; **, *p* < 0.01).

In this study, we first observed the influence of IL12RB1 deficiency on the in vivo containment of BCG. To mimic BCG vaccination and natural exposure to environmental mycobacteria, BCG was administered either subcutaneously (S.Q) or intranasally (I.N). After inoculation, the mice were weighed regularly. We observed a transient weight loss in wild-type mice on day 3, while the body masses of *Il12rb1*^−/−^ mice increased steadily, until the end of experiment (Figure S1). CFU counts showed that BCG inoculated subcutaneously was almost totally cleared in both the lungs and spleens of wild-type mice, while the bacterial loads in *Il12rb1*^−/−^ mice stayed at high levels (Figure 1a,b). For the mice being inoculated intranasally with BCG, significantly higher bacterial loads were also observed in the spleens of *Il12rb1*^−/−^ mice (Figure 1d). The average BCG counts in the lungs of *Il12rb1*^−/−^ mice after intranasal inoculation tended to be higher than that of wild-type mice, but they did not reach statistical significance (Figure 1c). We noted that the CFU counts were low in few intranasally inoculated IL12RB1-deficient mice, which was probably due to the natural protection against BCG infection in the respiratory tract.

### 3.2. Profiling of Cytokine Transcription in Lung Tissue Revealed Significantly Altered Innate Responses upon BCG Inoculation in Il12rb1^−/−^ Mice

**Figure 2:**
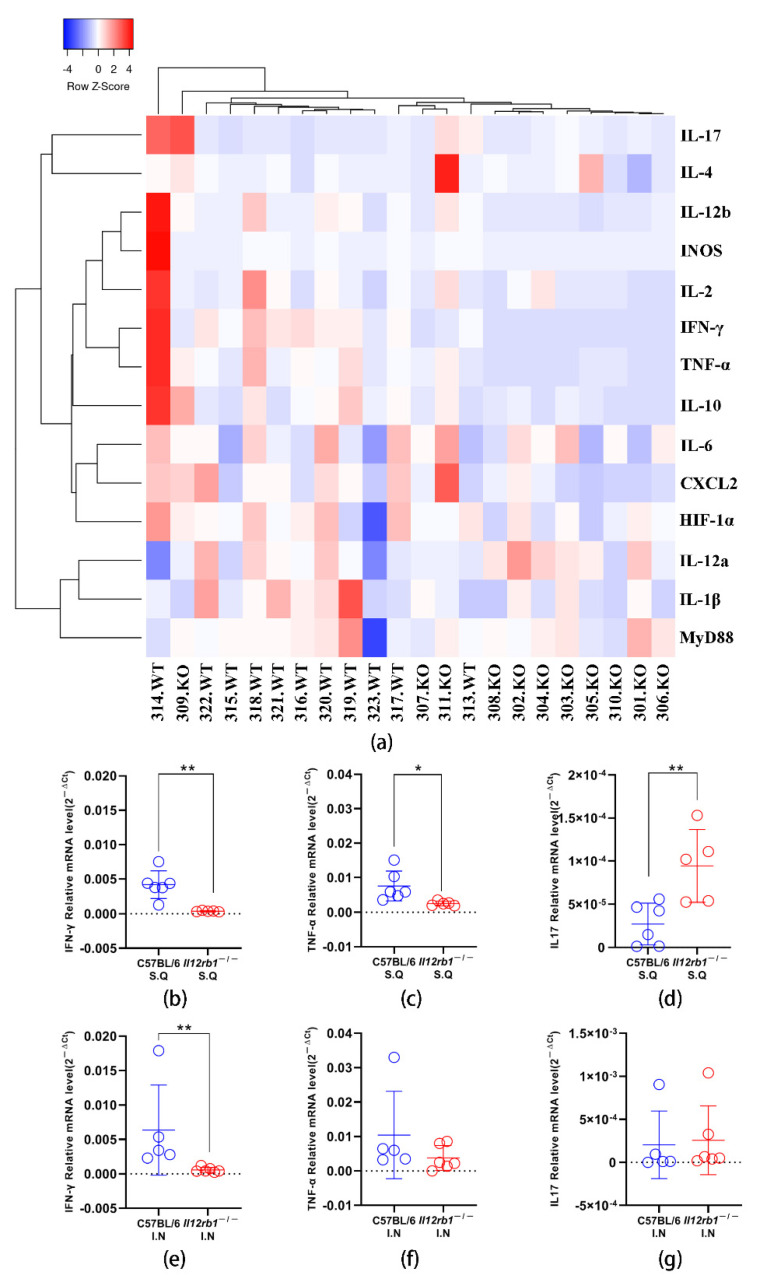
Hierarchical clustering analysis for transcriptions of multiple innate factors in lung tissue and cytokines differentially transcribed in lung tissues of *Il12rb1*^−/−^ and C57BL/6 mice after BCG inoculation. Transcription levels of 14 innate factors in lung tissues of *Il12rb1*^−/−^ (*n* = 11) and C57BL/6 mice (*n* = 11) were measured by real time PCR. And the general response patterns were visualized by heatmap clustering analysis (**a**). The transcription levels of IFN-γ (**b**,**e**), TNF-a (**c**,**f**), and IL-17A (**d**,**g**) in lung tissues were compared between *Il12rb1*^−/−^ and C57BL/6 mice after being inoculated with BCG, either subcutaneously or intranasally. Statistical analyses were performed by the two-tailed *t*-test method (*, *p* < 0.05, **, *p* < 0.01).

To gain insight into the influence of IL12RB1 deficiency on innate immune responses against BCG, total RNA was extracted from the lung tissue of each mouse, and the transcription levels of 14 innate factors were analyzed by qPCR. Our data showed that the innate immune responses of *Il12rb1*^−/−^ mice differed obviously from those of wild-type mice (Figure 2a and Figure S2). The heatmap clustering discriminated most of the wild-type mice from the *Il12rb1*^−/−^ mice, except one KO mouse (309.KO) and one WT mouse (313.WT). While responses triggered by different inoculation routes could not be clearly separated, either within the knockout or within the wild-type mice (Figure 2a). Of note, we found that the transcription levels of IFN-γ were significantly lower in *Il12rb1*^−/−^ mice, regardless of inoculation routes (Figure 2b,e). Moreover, our result showed that the transcription levels of TNF-a decreased, while the transcription levels of IL-17 increased in *Il12rb1*^−/−^ mice after being inoculated with BCG subcutaneously (Figure 2c,d). Similar patterns were also observed in intranasally inoculated *Il12rb1*^−/−^ mice, although the differences were not statistically significant (Figure 2f,g).

### 3.3. PPD-Specific T Cell Responses Were Partially Impaired in Il12rb1^−/−^ Mice

**Figure 3:**
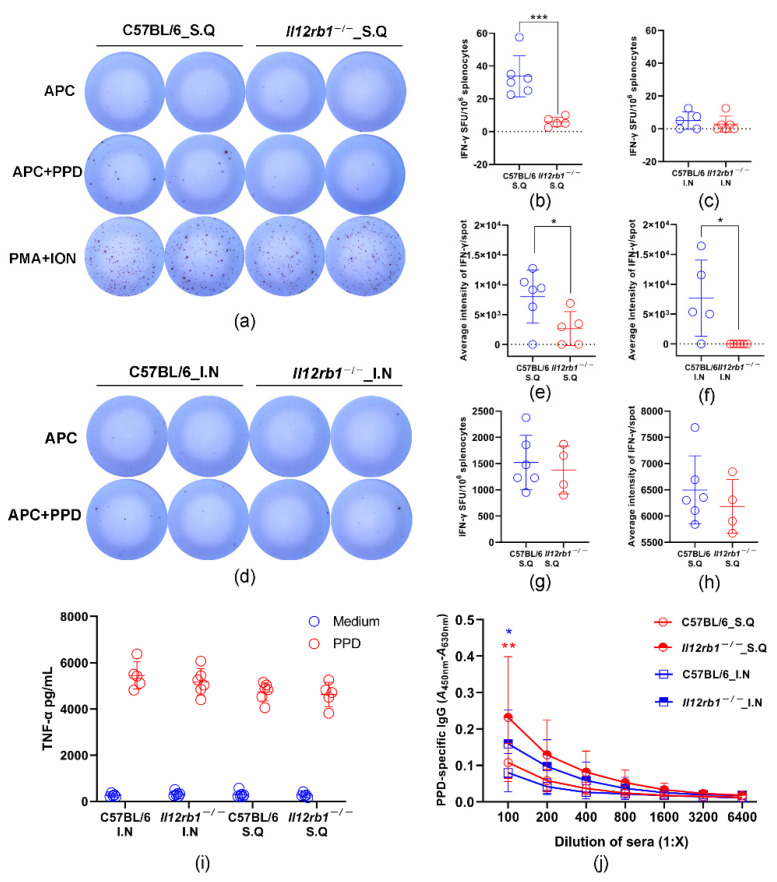
The evaluation of PPD-specific immune response. Peritoneal macrophages were added to ELISPOT plates to enhance antigen presentation. (**a**,**d**) Representative images of spots formed after being stimulated with APC only, APC+PPD, or PMA+ionomycin. Numbers of PPD-specific IFN-γ secreting cell were compared between *Il12rb1*^−/−^ and C57BL/6 mice after being inoculated with BCG, either subcutaneously (**b**) or intranasally (**c**). The comparisons of the average optical intensities of spots stimulated by PPD between *Il12rb1*^−/−^ and C57BL/6 mice after being inoculated with BCG, either subcutaneously (**e**) or intranasally (**f**). (**g**,**h**) showed the comparisons of IFN-γ secreting cell numbers and the average optical intensities of spots between *Il12rb1*^−/−^ and C57BL/6 mice after PMA+ionomycin stimulation. (**i**) The cell culture supernatant in the wells of ELISPOT plates was collected for CBA assay. Levels of TNF-a were compared between *Il12rb1*^−/−^ and C57BL/6 mice inoculated with BCG, either subcutaneously or intranasally. All the above data were shown as mean ± SD. Statistical analysis were performed by the method two-tailed *t*-test (*, *p* < 0.05, **, *p* < 0.01, ***, *p* < 0.001). PPD-specific binding antibodies were detected by ELISA. Optical density (OD) values were compared between *Il12rb1*^−/−^ and C57BL/6 mice (**j**). Data are shown as mean ± SD. Statistical analyses were performed by the two-way ANOVA method (*, *p* < 0.05, **, *p* < 0.01).

To understand the influence of IL12RB1 deficiency on adaptive cellular immunities against BCG, we measured the PPD-specific T cell responses using an antigen presentation-improved ELISPOT assay. Our data showed that the numbers of PPD-specific IFN-γ secreting cells in spleens of *Il12rb1*^−/−^ mice was lower than those of wild-type mice (Figure 3a–d), especially when BCG was inoculated subcutaneously (Figure 3b, *p* = 0.001). In addition to counting the number of spots forming cells, we also analyzed the optical intensities of spots, which reflect the quantities of IFN-γ secreted by T cells. Our data showed the average intensities of the IFN-γ spots formed after PPD stimulation were significantly lower in *Il12rb1*^−/−^ mice than those in wild-type mice (Figure 3e,f). In contrast to PPD stimulation, both the numbers and optical intensities of the spots generated after PMA/ionomycin stimulation showed no significant difference between *Il12rb1*^−/−^ and wild-type mice (Figure 3g,h). To further characterize the PPD-specific cellular immune responses, we analyzed the concentrations of TNF-a, IL-2, IL-17A, and IL-21 secreted into the culture supernatant during IFN-γ ELISPOT assay using a CBA kit. Among the four cytokines tested, PPD-specific production was only observed for TNF-a. As being depicted in Figure 3i, the levels of secreted TNF-a were comparable between the *Il12rb1*^−/−^ and wild-type mice.

### 3.4. PPD-Specific Antibody Responses Were Significantly Enhanced in Il12rb1^−/−^ Mice

PPD-specific antibodies in mouse serum were detected by ELISA. As shown in Figure 3j, *Il12rb1*^−/−^ mice exhibited stronger binding antibody responses against PPD than wild-type mice in both the subcutaneous and intranasal inoculation groups. We also evaluated the antibody avidity by the method of avidity ELISA assay, which showed that the antibody avidity levels were similar between the *Il12rb1*^−/−^ and wild-type mice (Figure S3).

### 3.5. IL-12a Correlated with Better Containment of BCG in Il12rb1^−/−^ Mice

**Figure 4:**
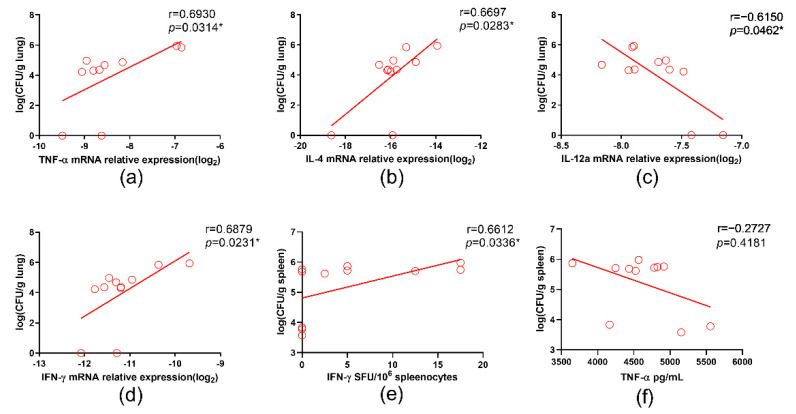
Correlation analyses BCG CFU counts and responses of multiple cytokines in *Il12rb1*^−/−^ mice. Transcription levels of TNF-a (**a**), IL-4 (**b**), IL-12a (**c**), and IFN-γ (**d**) correlated significantly with CFU counts in lung tissue. PPD-specific IFN-γ responses correlated positively with CFU counts in spleen (**e**), while no significant correlation was observed between PPD-specific TNF-a response and CFU counts in spleen (**f**). Normally distributed data was analyzed by the method of Pearson correlation, otherwise Spearman’s correlation was used (*, *p* < 0.05).

To identify the immune factors that potentially contributed to the in vivo control of BCG in *Il12rb1*^−/−^ mice, we performed correlation analyses among multiple immune factors and CFU counts. Our results showed that the transcription levels of the TNF-a, IL-4, and IFN-γ positively correlated with CFU counts in lung tissues (Figure 4a,b,d), while IL-12a showed a remarkable negative correlation with pulmonary BCG counts (Figure 4c). Consistent with the observation in lung tissues, the number of PPD-specific IFN-γ secreting splenocytes also positively correlated with the BCG counts in spleen (Figure 4e). There was a trend of negative correlation between the PPD-specific TNF-a release and BCG counts in the spleen, but it was not statistically significant (*p* = 0.4181) (Figure 4f). Other factors, such as the transcription levels of IL-1, IL-2, CXCL-2, Myd88, IL-17, IL-6, IL-12b, IL-10, INOS, and HIF-1a, did not correlate significantly with the CFU counts in the lungs (data not shown). PPD-specific IgG did not correlate with the bacterial loads either (Figure S4a,b). Correlation analyses were also performed between the innate factors and CFU counts in the wild-type mice; significant correlations were observed between the TNF-a, IL-2, IL-10, IL-17, HIF-1a, and INOS mRNA levels and CFU counts in the lungs (Figure S5).

Correlation analyses between the transcription levels of various factors in lungs showed that significant correlations widely existed between different factors (Figure S6). Of note, IL-12b correlated with IL-6, TNF-a, IL-2, IL-10, CXCL-2, IFN-γ, HIF-1a, and INOS, while IL-12a only correlated with TNF-a.

## 4. Discussion

IL12RB1 is the common chain shared by the IL-12 and IL-23 receptors [25,26]. The deficiency of IL12RB1 impairs IFN-γ immunity against mycobacteria by abolishing the functions of both IL-12 and IL-23 [27,28], which leads to increased susceptibility to weak pathogenic or environmental mycobacteria. Cumulative evidence showed that IL12RB1 deficiency can cause disseminated BCG infection (BCGosis) [16,17,29,30]. Even though hundreds of IL12RB1-related MSMD kindreds have been reported worldwide [5,16,17,31,32,33,34], the incomplete clinical penetrance of IL12RB1 deficiency and low recurrence rate of mycobacteria infection [16,27] suggest that protective immunity still exists in these individuals. Previous studies investigated the association between single nucleotide polymorphism in the IL-12R gene and vaccine immunity [35,36]. However, the impact of IL12RB1 deficiency on innate and adaptive immunities against BCG has not been adequately elucidated. Aiming to fill this gap, we characterized the innate and adaptive immunities upon BCG inoculation in *Il12rb1*^−/−^ mice by comparing with wild-type mice. We found that *Il12rb1* knockout attenuated the innate immune responses upon BCG inoculation, either intranasally or subcutaneously, among which, the transcriptions of IFN-γ and TNF-a were significantly reduced, while transcription of IL-17 increased in the lung tissues of *Il12rb1*^−/−^ mice. The enhanced IL-17 response was unexpected and inconsistent with previous reports, showing that Th17 response was impaired by IL12RB1 deficiency [14,27,37,38]. We cannot exclude the potential impairment of IL12RB1 deficiency on Th17 mediated immune responses, because the transcription of IL-17 was measured using total RNA extracted from lung tissue in this study, which might obscure the potential difference in Th17 responses. The observed enhancement of IL-17 transcription might derive from an IL-23 independent resource [39]. In addition, multiple previous studies suggest that neutrophils also contribute to IL-17 production [40,41,42,43,44,45,46], but it is unlikely that observed enhancement of IL-17 transcription was derived from neutrophils in IL12RB1-deficient mice because available evidence indicates that the IL-12 signaling is essential for neutrophil function [47,48,49].

BCG-specific IFN-γ release was also found to be significantly tempered by *Il12rb1* deletion in this study. However, the intrinsic capability of T cells to produce IFN-γ might remain intact, because the secretion of IFN-γ was not hampered when stimulated with PMA and ionomycin. Moreover, our results also showed that BCG-specific TNF-a release was not impaired in *Il12rb1*^−/−^ mice, despite the reduced innate TNF-a response observed in the lungs, reflecting that IL-12 may utilize different mechanisms to regulate innate and adaptive immunities [50,51]. In addition to cellular responses, we also found that the BCG-specific binding antibody responses increased significantly. This observation was consistent with a previous study [15], which implied that the immune system might upregulate antibody response in compensation for the loss of cellular immune function.

Furthermore, in order to identify potential protective immune factors under the genetic setting of IL12RB1 deficiency, we performed correlation analyses between the CFU counts of BCG and multiple parameters. Unexpectedly, we found that both the innate and BCG-specific IFN-γ responses correlated positively with CFU counts, whereas the innate IL-12a response was shown to be negatively associated with CFU count in lung tissue. Supplementation of IFN-γ represents an attractive therapy for IL12RB1-deficient MSMD patients [52]. Nonetheless, our data suggests that IFN-γ did not help to control mycobacterial infection (Figure S4c,d), which is supported by a clinical report showing that IFN-γ treatment did not reduce serum mycobacterial activity [53]. Alternatively, our result indicated that IL-12a was negatively associated with CFU counts in IL12RB1 knockout mice, which suggested that it might benefit the containment of disseminated BCG infection for IL12RB1-deficient patients. We explored the therapeutic effect of IL-12a by supplementing the IL12RB1-deficient mice with it; however, the BCG loads were not significantly reduced (data not shown). We speculated that IL-12a might be necessary, but insufficient, for the control of BCG in these mice. As the genetic background of BCG is different from other non-attenuated mycobacteria, the characteristics of in vivo dissemination might be different. However, the patterns of host immune responses might be similar. Therefore, we speculate that the above conclusions might also be applied to other mycobacterial infections.

Several limitations should be noted for this study. First, the BCG load and immune responses were not dynamically monitored, which made it impossible to compare the compacities of wild-type and *Il12rb1*^−/−^ mice in controlling BCG infection at a time point either earlier or later than 4 weeks post infection. Second, as the PPD-specific total T cell responses were measured using the method of IFN-γ ELISPOT and CBA assays, the specific CD4 and CD8 T cell responses could not be evaluated separately. Fourth, the mechanism underlying the negative correlation between the IL-12a and BCG CFU counts is not clarified in the current study. Further investigation into these issues may shed new light on the function of IL-12 signaling in anti-mycobacteria immunity and find a new therapy for IL12RB1 caused MSMD.

## 5. Conclusions

The present study proved that, while the general innate immune response decreased, the adaptive immunities (including IFN-γ responses) against mycobacteria were not completely nullified in *Il12rb1*^−/−^ mice. More importantly, our results implied that IFN-γ responses alone might not be able to contain BCGitis in the setting of IL12RB1 deficiency.

## Figures and Tables

**Table 1:** The design of mouse experiment.

Group	Mouse Strain	BCG Dosage (CFUs/Mouse)	Inoculation Route	Schedule	
				Inoculation	Euthanasia
1	C57BL/6 (*n* = 5)	1.5 × 10^6^	Intranasally	Day 0	Day 27
2	*Il12rb1*^−/−^ (*n* = 6)				
3	C57BL/6 (*n* = 6)	3 × 10^6^	Subcutaneously		
4	*Il12rb1*^−/−^ (*n* = 5)				

## Data Availability

The datasets generated for this study are available on request to the corresponding author.

## Supplementary Materials

The following are available online at https://www.mdpi.com/article/10.3390/vaccines10071147/s1. Figure S1: Kinetics of body weight change after BCG inoculation. Figure S2: qPCR analysis for transcriptions of multiple innate factors in lung tissue of *Il12rb1*^−/−^ and C57BL/6 mice after BCG inoculation. Figure S3: Avidity analyses of PPD-specific antibodies in mouse serum. Figure S4: Correlation analyses between BCG CFU counts and PPD-specific IFN-γ or antibody responses. Figure S5: Correlation between BCG CFU counts and responses of multiple cytokines in C57BL/6 mice. Figure S6. Correlation analyses among the transcription levels of factors in lungs of IL12RB1-deficient mice. Correlations between normally distributed data were analyzed by the method of Pearson correlation, otherwise Spearman’s correlation was used. Table S1: Primers for qPCR assay of mouse cytokines.
