# Supplementary material for: Immune Correlates of Disseminated BCG Infection in IL12RB1-Deficient Mice
Source: Vaccines (Basel). 2022 Jul 19;10(7):1147. doi: 10.3390/vaccines10071147 (PMC9316795; doi:10.3390/vaccines10071147)
Supplement: Supplementary file 1 [file vaccines-10-01147-s001.zip › vaccines-1771263-supplementary.pdf]

vaccines

MDPI

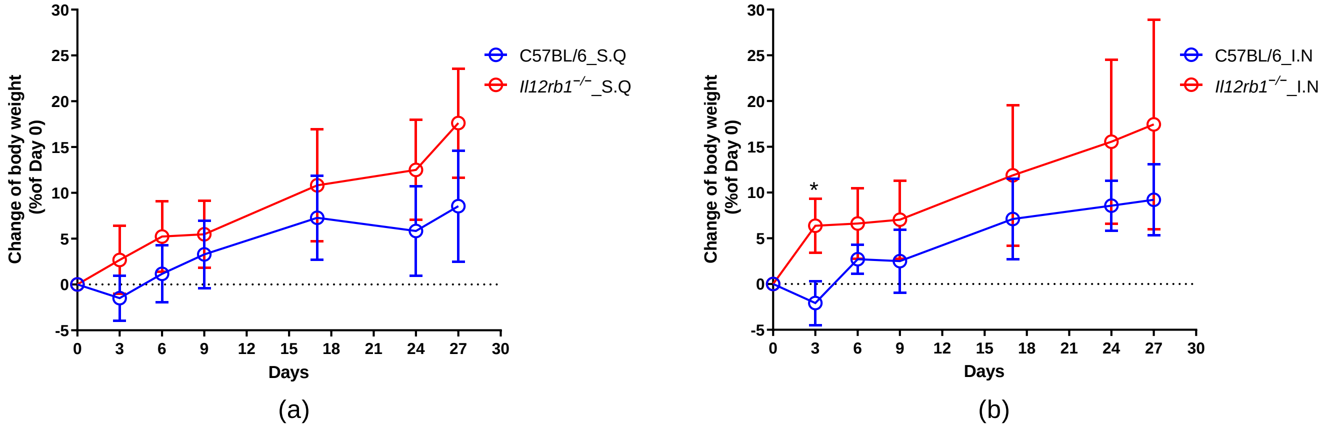

Figure S1. Kinetics of body weight change after BCG inoculation. (**a**) Body weight changes of mice being inoculated with BCG subcutaneously. (**b**) Body weight changes of mice being inoculated with BCG intranasally. Data are shown as mean ± SD (*, $p < 0.05$).

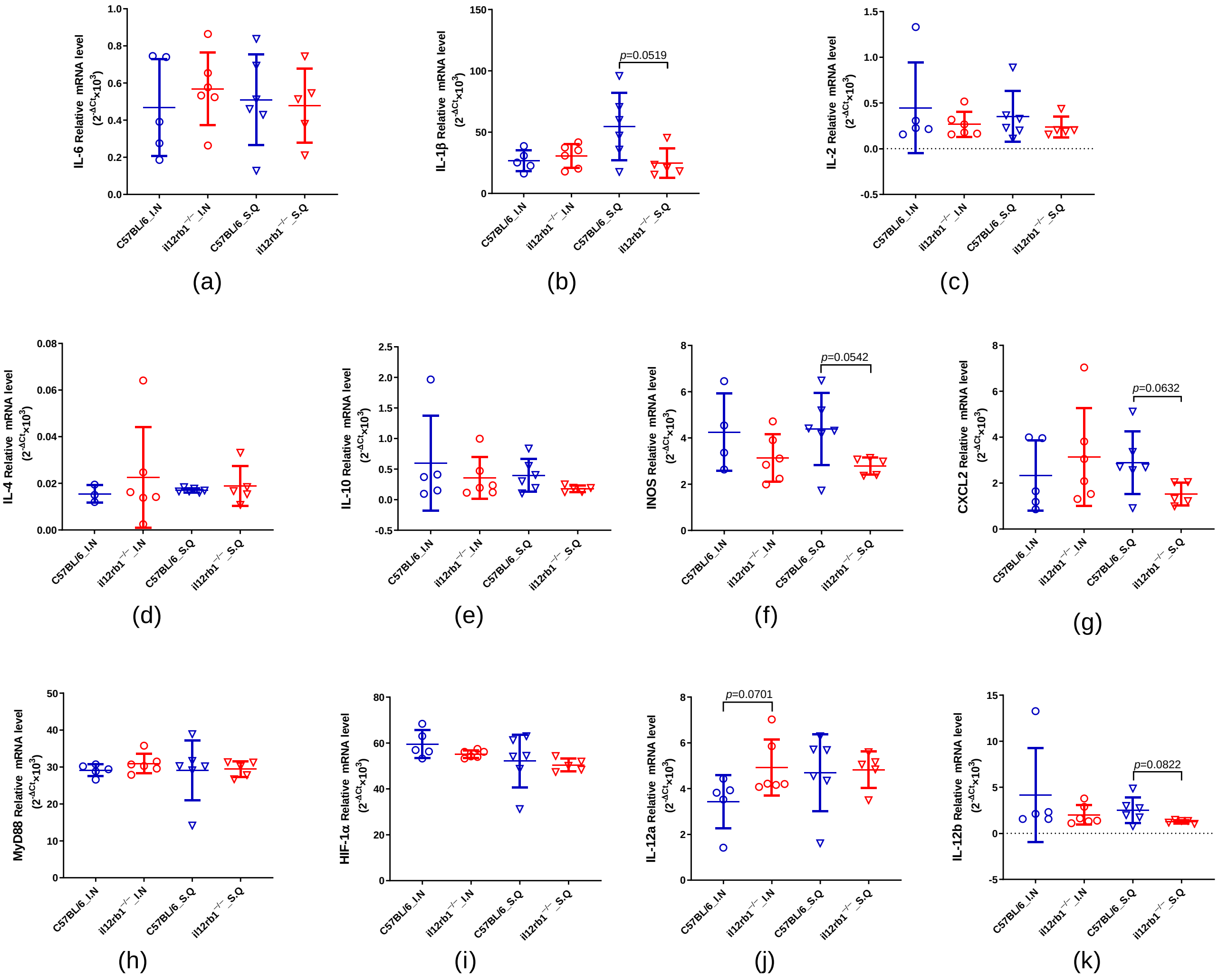

Figure S2. qPCR analysis for transcriptions of multiple innate factors in lung tissue of *Il12rb1*$^{-/-}$ and C57BL/6 mice after BCG inoculation. Transcription levels of 11 innate factors in lung tissues was

measured by real time PCR in *Il12rb1*$^{-/-}$ and C57BL/6 mice after being inoculated with BCG either subcutaneously or intranasally. (**a**) IL-6. (**b**) IL-1β. (**c**) IL-2. (**d**) IL-4. (**e**) IL-10. (**f**) INOS. (**g**) CXCL2. (**h**) MyD88. (**i**) HIF-1a. (**j**) IL-12a. (**k**) IL-12b. Statistical analysis were performed by the method of two-tailed *t-test*.

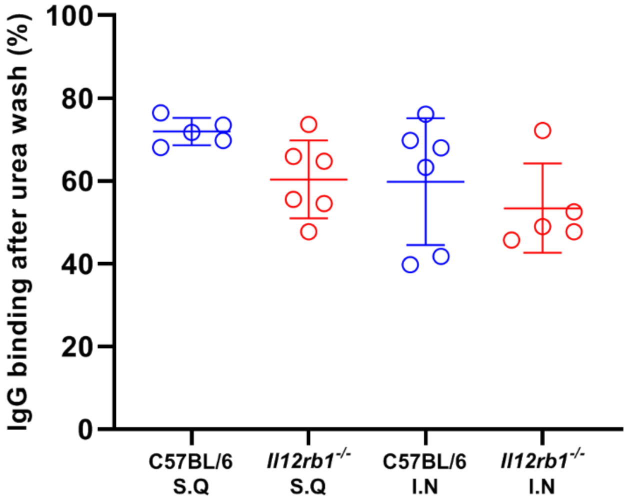

Figure S3. Avidity analyses of PPD specific antibodies in mouse **serum**. The avidities of PPD specific IgG were evaluated by ELISA. Briefly, after the binding of serum antibodies, the PPD coated plates were treated with 6M urea. The ratios of residual "high avidity" antibodies were calculated according to the following formula: (Absorbance after urea treatment /absorbance after PBS treatment × 100%). Data were shown as mean ± SD. Statistical analysis were performed by the method two-tailed *t-test*.

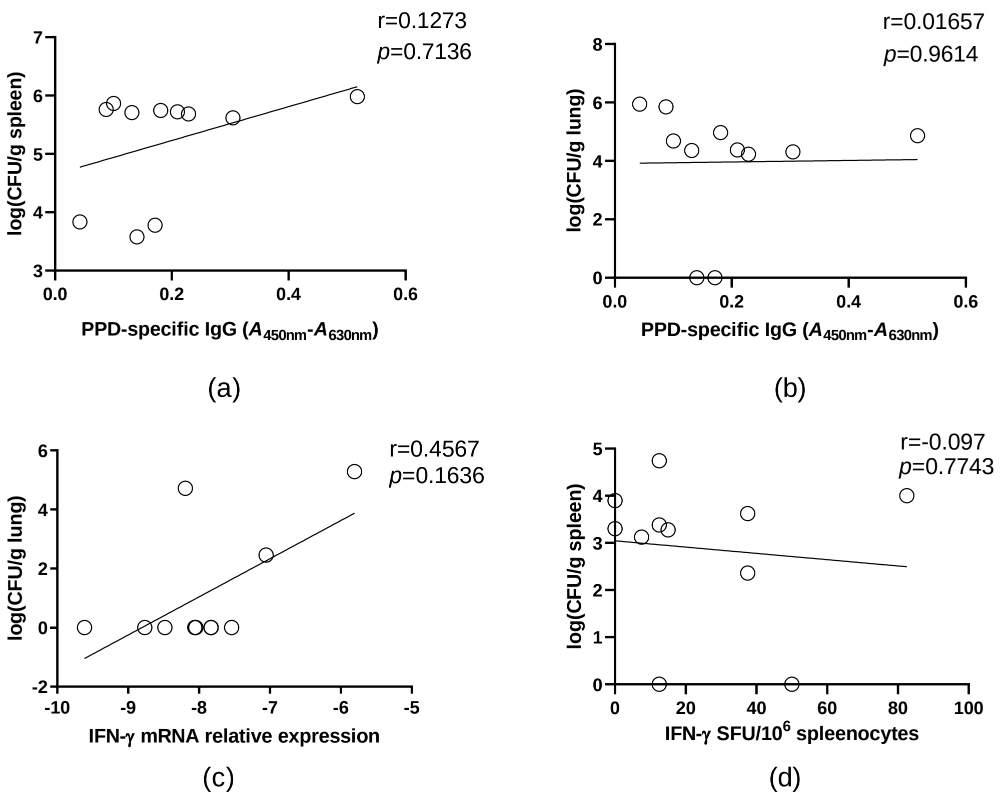

Figure S4. Correlation analyses between BCG CFU counts and PPD specific IFN-γ or antibody responses. (**a**) and (**b**) Correlations between PPD specific IgG levels with CFU counts of spleen or lung tissues in Il12rb1$^{-/-}$ mice. (**c**) Correlation analysis innate IFN-γ responses and CFU counts in lung tissues of wild-type mice. (**d**) Correlation analysis of PPD specific IFN-γ responses and CFU counts in spleens of wild-type mice. Correlations between bacterial burden and antibody or IFN-γ responses were evaluated using Spearman's correlation.

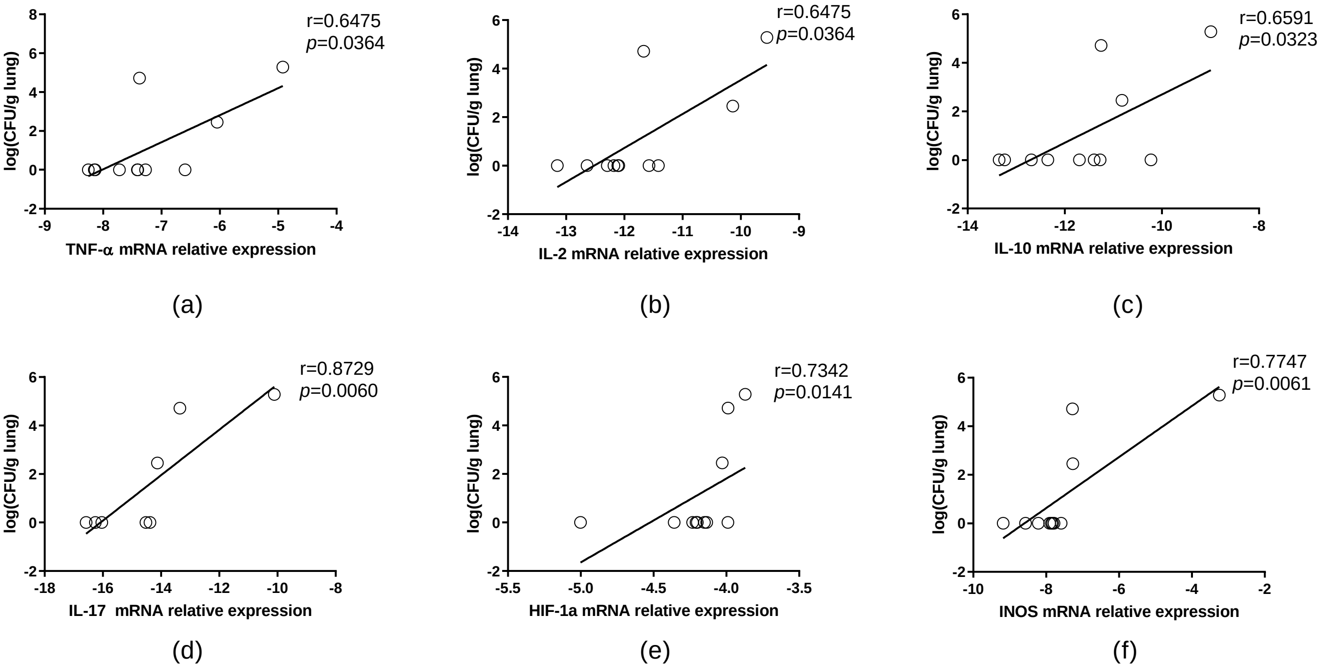

Figure S5. Correlation between BCG CFU counts and responses of multiple cytokines in C57BL/6 mice. Correlation of transcription levels of TNF-a (**a**), IL-2 (**b**), IL-10 (**c**), IL-17 (**d**), HIF-1a (**e**) and INOS (**f**) with CFU counts in lung tissue. Normally distributed data was analyzed by the method of Pearson correlation, otherwise Spearman`s correlation was used.

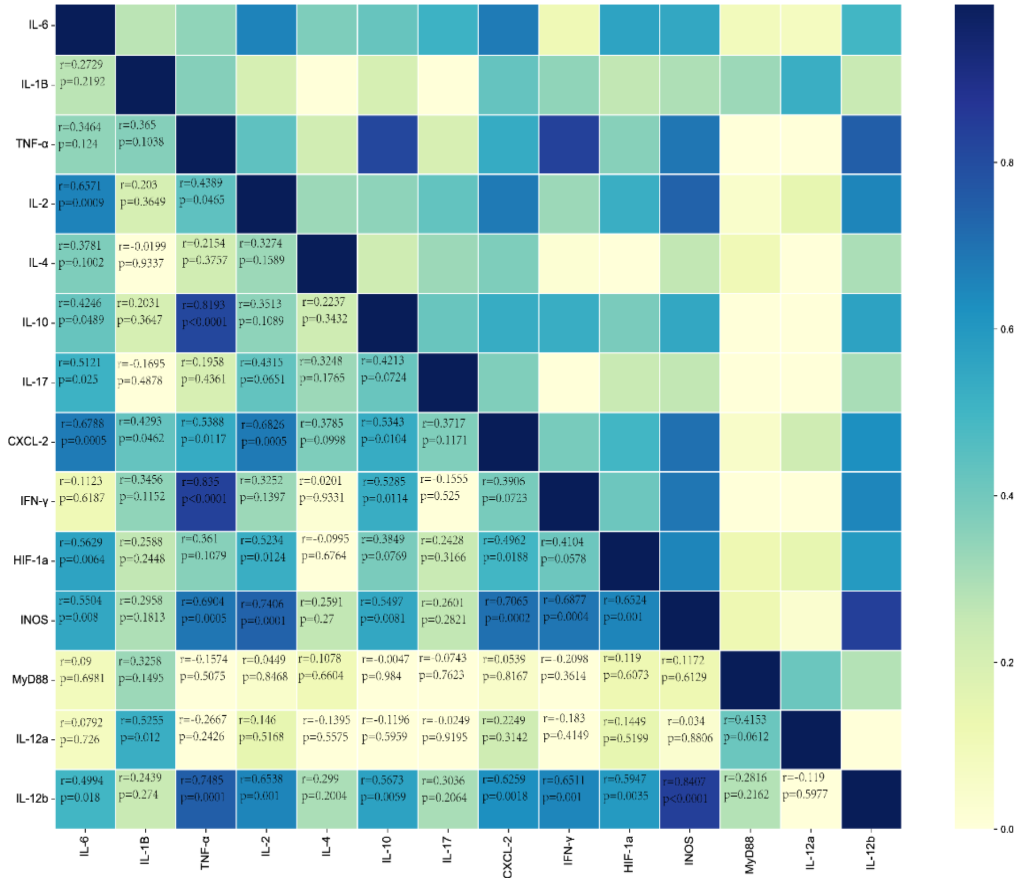

Figure S6. Correlation analyses among the transcription levels of factors in lungs of IL12RB1-deficient mice. Correlations between normally distributed data were analyzed by the method of Pearson correlation, otherwise Spearman`s correlation was used.

Table S1. Primers for qPCR assay of mouse cytokines.

Gene	Primer Sequence	Amplicon Length (bp)
TNF-α	F: CATCTTCTCAAAATTCGAGTGACAA<br>R: TGGGAGTAGACAAGGTACAACCC	175
IL-1	F: CAACCAACAAGTGATATTCTCCATG<br>R: GATCCACACTCTCCAGCTGCA	152
IL-2	F: TCTGCGGCATGTTCTGGATTT<br>R: ATGTGTTGTCAGAGCCCTTTAG	116
CXCL2	F: CCTGCCAAGGGTTGACTTCA<br>R: TTCTGTCTGGGCGCAGTG	76
Myd88	F: AGTGTCTGGGGGAGGAATGA<br>R: ACACTTGACCCAGGTTGCTT	94
IFN-γ	F: ACAGCAAGGCGAAAAAGGATG<br>R: ACAGCAAGGCGAAAAAGGATG	106
IL-17a	F: GGCCCTCAGACTACCTCAAC<br>R: TCTCGACCCTGAAAGTGAAGG	225
IL-4	F: ATCGGCATTTTGAACGAGGTC<br>R: GAGGACGTTTGGCACATCCA	69
IL-6	F: GAGGATACCACTCCCAACAGACC<br>R: AAGTGCATCATCGTTGTTCATACA	141
IL-12a	F: CTGTGCCTTGGTAGCATCTATG	170

	R: CGCAGAGTCTCGCCATTATGAT	
Il-12b	F: CTCAGAAGCTAACCATCTCCTGG<br>R: CACAGGTGAGGTTCACTGTTTC	144
IL-10	F: AAGCCTTATCGGAAATGATCCA<br>R: GCTCCACTGCCTTGCTCTTATT	180
INOS	F: CCATCATGAACCCCAAGAGT<br>R: CTGGCCAGATGTTCCTCTAT	142
HIF-1α	F: ACCTTCATCGGAAACTCCAAAG<br>R: ACTGTTAGGCTCAGGTGAACT	187
GAPDH	F: ACCCAGAAGACTGTGGATGG<br>R: GGATGCAGGGATGATGTTCT	81
